# Using Mice to Model Human Disease: Understanding the Roles of Baseline Housing-Induced and Experimentally Imposed Stresses in Animal Welfare and Experimental Reproducibility

**DOI:** 10.3390/ani12030371

**Published:** 2022-02-03

**Authors:** Bonnie L. Hylander, Elizabeth A. Repasky, Sandra Sexton

**Affiliations:** 1 Department of Immunology, Roswell Park Comprehensive Cancer Center, Buffalo, NY 14263, USA; elizabeth.repasky@roswellpark.org; 2 Laboratory Animal Shared Resources, Roswell Park Comprehensive Cancer Center, Buffalo, NY 14263, USA

**Keywords:** mouse models, reproducibility, chronic stress, housing temperature, animal welfare

## Abstract

**Simple Summary:**

Scientific research into the causes, progression, and treatment of disease is dependent on the use of animal models. However, many scientists say that they cannot repeat published experiments. Studies designed to investigate the scope of this problem have reported that less than half of the experiments could be successfully repeated. Some reasons are the incomplete description of the experimental protocol, difficulties in identifying and obtaining the reagents and animals models used, and other issues in the actual experimental design and interpretation. However, another important facet of animal research that contributes to these differences is overall animal welfare, which directly impacts experimental outcomes. Mildly cool housing temperature causes chronic stress in mice and has been identified as a factor that can alter experimental outcomes, including experiments involving immunity. This review considers how chronic stress (inadvertently imposed on mice by housing conditions and intentionally by researchers studying effects of stress) both compromise animal welfare and consequently impact experimental outcomes. Increasing awareness of how differing levels of chronic stress can underlie different outcomes in similar experiments will improve animal welfare and experimental reproducibility and should also improve translatability of discoveries to the clinic.

**Abstract:**

Mice are the most common animal used to study disease, but there are real concerns about the reproducibility of many of these experiments. This review discusses how several different sources of chronic stress can directly impact experimental outcomes. Mandated housing conditions induce an underappreciated level of chronic stress but are not usually considered or reported as part of the experimental design. Since chronic stress plays a critical role in the development and progression of many somatic diseases including cancer, obesity, and auto-immune diseases, this baseline stress can directly affect outcomes of such experiments. To study the role of stress in both physical and psychiatric diseases, there has been a proliferation of protocols for imposing chronic stress on mice. For somatic diseases, biomarkers can be used to compare the models with the disease in patients, but to evaluate the validity of psychiatric models, behavioral tests are carried out to assess changes in behavior and these tests may themselves cause an underappreciated degree of additional stress. Therefore, it is important for animal welfare to reduce baseline stress and select the most humane protocols for inducing and assessing chronic stress to obtain the most reliable outcomes.

## 1. Introduction

Approval of new therapeutics for clinical use only follows lengthy clinical trials that ensure both the safety and efficacy of these potentially new therapeutic strategies in pre-selected, closely monitored groups of patients. However, the initiation of clinical trials is based on the outcomes of extensive preclinical mouse studies that form the basis for understanding the biology of the disease, host responses to disease, and the mechanism of action of the most promising lead therapies that are chosen to move forward into non-rodent studies and subsequent clinical trials [1,2,3]. This necessary dependence on animal models creates complex responsibilities that require researchers, care-givers, and advocates to thoughtfully evaluate every aspect of animal care and use. Thus, the creation of the Guide for the Care and Use of Laboratory Animals [4]. Implementation of the principles in the Guide is required in the United States by the Public Health Service Policy and its recommendations are made based on performance standards that are used as the foundation for the conditions under which research animals are housed, as well as standards for procedures performed on research animals. Creation of Institutional Animal Care and Use Committees (IACUC) is therefore required to oversee the details of experimental protocols. In the United States, the National Institutes of Health(NIH) and other granting agencies require institutions to provide assurance of compliance with The Guide’s standards as a condition for receiving funds. The NIH itself also has comprehensive measures in place, overseen by the Office of Laboratory Animal Welfare (OLAW), to ensure the humane treatment of animals in all NIH funded research projects. Facilities worldwide that are Association for Assessment and Accreditation of Laboratory Animal Care International (AAALAC International) accredited, often go above and beyond the animal research regulations required by law, focusing on the quality of research and the welfare of the animals. 

One of the key initiatives that has developed regarding animal use in research is the 3Rs (Replacement, Reduction, and Refinement) put forth in the early 1960s by two English biologists, Russell and Burch, in their book “The Principle of Humane Experimental Technique”. The National Centre for the Replacement, Refinement and Reduction of Animals in Research in the UK is an organization that supports researchers and others to advance and implement the 3Rs and, if animals must be used, to make researchers aware of how improving animal welfare improves the quality of the results obtained from experiments [5,6]. Inclusion of the concept of the three Rs in multinational laws, regulations, and guidelines gives these concepts significant influence over how global animal research is conducted today. However, the concepts put forth in the 3Rs are over 50 years old, and our ideas about the ethics for the treatment of research animals have changed as our understanding of animal cognition, behavior, and what is necessary for their welfare has changed; during this time, public sensibilities towards animals have also changed [7,8] and a new framework has been proposed based on balancing the principles of “social benefit and animal welfare” incorporating the idea that we should provide for animal welfare not just during experiments, but during their everyday lives in captivity. This is particularly timely as concern over reproducibility (and how lack of reproducibility affects the numbers of animals used in research) is receiving widespread attention [1,9]. (Sometimes a distinction is drawn between “reproducibility”, used to mean obtaining the same conclusion analyzing the same results by the same methods, and “replicability,” meaning to obtain the same results with new experiments and data, but here we are using reproducibility in the more general sense of being able to repeat an experiment and get confirmatory results.) Two studies which drew attention to this issue were by Bayer [2] and Amgen [10]. These concluded that less than 25% and 11%, respectively, of the studies attempted, were able to be repeated. Subsequently, Errington and colleagues undertook the “Reproducibility Project: Cancer Biology” and have recently published their results [11,12]. These authors were able to reproduce/replicate only a small percentage of the experiments they set out to study and identified lack of statistically relevant information, lack of access to code, lack of information about reagents, and lack of access to mouse/cell models or reagents as reasons. To improve reproducibility, Landis and colleagues [13] advocated for including a core set of parameters in publications, thereby providing adequate information about the experimental design to enable its reproduction. However, they acknowledged that even though this is an important step, it will take a “concerted effort by all stakeholders, including funding agencies and journals, … to disseminate and implement best reporting practices …”.

The ARRIVE Guidelines (Animal Research Reporting of In Vivo Experiments) were published in 2010 and attempted to address these information gaps in publications by creating a list of 20 factors that should be reported [14,15,16]. More recently, it was been pointed out that although this effort had broad support, the guidelines have not been widely followed and have not had the desired impact [17], leading to a revised version, ARRIVE 2.0, released in 2020 [18]. Refocusing the emphasis of the ARRIVE guidelines on an “Essential 10” should allow authors to provide the most important information that will enable readers to duplicate the experiments and we support this effort. Some journals now have a checklist to accompany submissions (e.g., the Nature family’s Reporting Summary to “improve the reproducibility of work that we publish”). One underlying reason for the current abbreviated reporting of experimental details are the word limits commonly imposed by journals to save space; the judicious use of supplementary materials could facilitate the inclusion of detailed descriptions of experimental design and methods of analysis that would otherwise be omitted [19]. However, it will still be up to investigators, journals, and funding agencies to ensure that the experimental details needed to reproduce the original experiment and obtain valid results are available. Such results may or may not support the same conclusions, but by being able to accurately reproduce an experiment, a consensus on the outcome can be reached by multiple labs. 

Inherent in the 3Rs is the concept that reducing the distress (i.e., “stress”) experienced by research animals will improve their welfare and thus the validity of experimental results [6,20,21]. As a sidenote, we often use the terms stress and distress interchangeably, but they are slightly different [22]. Stress refers to a perturbation in homeostasis and the “stress response” comprises the physiological and behavioral changes taken to re-establish homeostasis. When these responses can no longer cope with the stress, and the animal may be suffering or in pain, and if its welfare is severely compromised, the animal is said to be in distress. Clearly, an animal in distress is not going to provide reliable experimental data. Strikingly, it is also becoming clear that although animal welfare may not be obviously compromised by stress, chronic stress has significant potential to skew the outcomes of experiments but often is not recognized or controlled for. This is a major reason to identify and understand the multiple sources of stress experienced by research animals. Accounting for potential sources of stress when planning experiments and analyzing results will also support current efforts to improve experimental design [23] and can be incorporated into the PREPARE (Planning Research and Experimental Procedures on Animals: Recommendations for Excellence) guidelines [24] or the online Experimental Design Assistant [25] for help with planning experiments. 

## 2. Housing Conditions Cause Chronic, Inadvertent, Baseline Stress

The Guide has standardized parameters for housing variables that were chosen to provide for the well-being and overall health of research mice; however, the people tasked with implementing these guidelines are the animal caretakers. Researchers, if they consider these housing variables at all, largely presume that baseline housing guidelines were selected because they are optimal. Taking the baseline status of mice for granted, researchers overlay experimental designs on these mice without specifically considering the housing conditions. Recently, Barbee and Turner framed the effect of these “undefined” and unreported environmental housing conditions as an “uncontrolled vivarium experiment” that runs in parallel to the actual well designed, scientifically controlled experiment [26]. We and others have been concerned that many of these “behind the scenes” environmental choices inadvertently cause an unaccounted-for degree of chronic stress that significantly impacts experimental outcomes and is a source of variability between experiments. This viewpoint was expressed several years ago by the editors of Nature Neurobiology, who wrote: “Factors such as animal housing, handling, food, lighting, and noise conditions, all of which effect behavior and brain chemistry, can be varied. The key to reproducibility is accurate reporting of these seemingly mundane details, which potentially have large effects” [27]. Additionally, there is a more recent push to actively improve living conditions to encourage the natural behaviors of mice, rather than to just avoid physical harm [28]. Martin et al. [29] were among early advocates for acknowledging a fuller appreciation of the effects of housing on experimental outcomes by questioning the status of “control” mice, stating that “The use of overweight and unstimulated animals as standard controls may bias the measured experimental outcomes” pointing out the fact that lab mice have unlimited access to food, are maintained in unstimulating shoebox cages, and get little exercise. These conditions are the direct opposite of their natural environment. These authors discuss how overweight, sedentary rodents have increased inflammation, faster tumor incidence and growth, and metabolic risk factors for obesity, heart disease, and diabetes. They asked whether the failed promise of many therapeutics in the clinic is because they were tested in metabolically compromised animals and whether the therapies might be acting on the underlying metabolic conditions associated with an unhealthy lifestyle rather than the disease itself. Furthermore, these authors advocated for testing therapies in two cohorts of mice housed under different conditions. 

As stated in a more recent review article by Toth, although researchers recognize that it is important to maintain good animal welfare, they are overall unaware of “how significantly even seemingly minor variations in the environment can affect research outcomes” [9]. As an example, the author discusses the differences in basic housing conditions (temperature, density, caging systems, and bedding) that were reported for research mice being used to study the effect of one gene on several parameters including sleep, immune response, tumor growth, inflammation, and infections. Toth points out how these different housing conditions between sites could induce physiological differences that make cross-study comparisons problematic. Other relevant factors include differences in cage type and rack position, color of cage and light, types of bedding, ambient temperature, noise from a variety of sources, whether mice are housed separately or socially, the progressive removal of mice during experiments, types of food and water [30], handling [31,32], and variable types of environmental enrichment [33] all can have subtle effects on the physiology and behavior of mice that can impact experimental outcomes [9,34]. A common denominator involved in these subtle differences in physiology and behavior is that choices in implantation can subject mice to chronic stress. 

One factor in this stress is the small barren cages in which mice are kept and one solution to reducing the psycho-social stress and encouraging natural behaviors has been to provide a variety of environmental enrichment (EE) devices in larger cages to create a more interesting, engaging environment for the mice, which encourages more activity and interactions [9]. There are many possibilities for EE devices including shelter huts, exercise equipment, novel objects for exploration, cotton balls and nesting material, and even cage mates can be considered EE but every addition alters the stress levels, may disrupt or intensify social status issues, and variability may cause significant fluctuations in experimental outcomes [9]. One approach to reduce variability in EE effects is proposed by Slater and Cao [35], who suggest that inconsistency in EE implementation results in lack of reproducibility of EE effects and published a protocol for standardization of EE; they recommend specific EE devices including plastic tubing, igloos/huts, metal running wheels, and small wooden logs (which should be moved around to provide novelty) and include specific cage sizes for the home cage with a hole for mice to enter the enrichment bin. These authors have shown that compared to mice in shoebox caging, mice in EE have decreased adipose tissue, increased energy expenditure, resistance to diet induced obesity, and slowed B16 melanoma tumor growth in C57BL/6 mice [35]. Others have also reported reduced tumor growth when mice are housed under EE conditions. Li et al. [36] found that the combination of EE devices (huts, tunnels, wooden toys, nesting material and running wheels), in conjunction with large cohorts of mice (12) housed in the large EE cage reduced pancreatic tumor growth in C57BL/6 mice, whereas running, EE devices, or large social cohorts alone achieved the same degree of tumor inhibition. Similarly, an EE model consisting of running wheel, tunnels, igloos, nesting material and wooden toys, with 10 mice/cage resulted in significant inhibition of breast tumor growth in an orthotopic model in C57BL/6 mice. Interestingly, in a model of minimal EE (mEE) using ovarian tumors implanted into B6C3F1 mice (a hybrid strain with a male C3H and a female C57BL/6 mouse), mEE (a hut) for 6 weeks prior to tumor implantation, mice activated NK cells and inhibited tumor growth, whereas if mice were not acclimated to mEE prior to tumor implantation, no benefit was seen [37]. 

An important source of chronic stress that has received particular attention over the last decade is the ambient temperature at which mice are housed. The mandated temperatures (20–26 °C) are known to be below the thermoneutral temperature for mice (~30 °C) [38], but are a compromise made in deference to the thermal comfort of the personnel who work in the mouse facilities [39]. Although the welfare of mice housed at sub-thermoneutral temperatures does not appear to be compromised, these mice are chronically cold-stressed and activate non-shivering thermogenesis to maintain normal body temperature of ~37 °C. An early and clear demonstration of how this elevated energy use affects experimental outcome was reported by Feldman’s group who showed that a genetically engineered mouse model (with Uncoupling Protein 1,UPC1, ablation) did not show the expected obese phenotype when mice were housed under standard temperatures, but mice did become obese when housed at thermoneutrality [40]. Several investigators have expressed grave concerns about the metabolic differences in mice housed at standard vs. thermoneutral temperatures, concluding that under cool housing, mice have “increased food intake, metabolic rate, sympathetic activity (which drives thermogenesis), blood pressure and heart rate [41,42,43]. Norepinephrine (NE) is released from sympathetic nerve terminals during the sympathetic stress response and binds to adrenergic receptors present on essentially all cells (parenchymal, immune, other stromal cells, and tumor cells). We have demonstrated that NE levels are higher when mice are housed at the cooler, standard temperature of 22 °C (without additional nesting materials) than at thermoneutrality 30 °C [44,45]. Additionally, Uchida et al. [46] compared metabolic parameters of mice housed at mildly cool 20 °C and mice housed at “near-thermoneutrality” 25 °C and found a significant elevation in serum NE in mice at the cooler temperature.

In addition to its effects on obesity models, housing mice at thermoneutrality vs. standard temperature has been shown to change the outcome in experiments using mouse models of nonalcoholic fatty liver disease [47], asthma [48], oral antigen sensitization [49], obesity and inflammation [50], osteoporosis [51], infection [52], atherosclerosis [53], and immune responses (LPS) [50]. Our lab has reported that cool ambient temperature, and the resultant adrenergic stress response, promotes tumor growth [45,54,55], reduces response to chemotherapy and radiation [56,57] and immunotherapy [44], reduces the anti-tumor immune response [44,58], immune cell function [59,60,61,62], graft vs. host disease [63], and heat shock protein induction [64]. We have also found elevated levels of NE in blood and tumors of mice housed at standard temperature [44,45]. That many of the observed differences in responses are driven by adrenergic signaling (particularly through beta-adrenergic receptors, β-ARs) is demonstrated by the fact that blockade of adrenergic signaling with “β-blockers” mitigates the housing temperature mediated differences [44,45,61,62,63,65]. Others have proposed approaches by which mice could mitigate this cold stress, such as nest building [66,67]. We have previously reviewed this topic [68,69,70]. Like nest building, mice in the wild can self-select the temperature at which they are comfortable at any given time. To recapitulate this opportunity in research mice, our group designed and tested a caging system in which a cavity under the floor allows for daily replacement of a chemical “hand-warmer” [71]. Tumor-bearing mice housed in the cages with a localized heat source showed better tumor control and beneficial changes in immune contexture that were comparable to those of mice housed at thermoneutrality compared to mice housed at standard temperatures [72] These observations suggest that at least some aspects of the lack of reproducibility in rodent research may be related to variables in housing, husbandry, and thermoregulation of animals [73], but that these effects can be identified and mitigated. 

Included in housing/husbandry choices that are underappreciated sources of stress for laboratory animals is the method used for handling the mice. Commonly, mice are picked up by grasping the base of the tail; however, this induces stress and creates anxiety [74]. Alternatively, non-aversive methods that have been studied include “tunneling”, in which a mouse is encouraged to enter a plexiglass tube kept in the home cage and then the tube is lifted out, and “cupping”, in which mice are gently scooped into a gloved palm and then lifted out of the cage. In both cases, physical restraint is avoided and mice are amenable to further procedures such as gavage or injection, without the added fear and stress of being abruptly “captured” [74]. Ghosal et al. [75] showed that picking mice up by the tail (vs. cupping) increased both physiological measurements of stress (elevated blood glucose and corticosterone levels) and anxiety-like behaviors in assessments of anxiety (i.e., elevated plus maze).

A particularly stressful time for research mice is during transportation and introduction of animals into a new housing facility. This abrupt change in environment may have detrimental effects on the animals’ general health and well-being. Therefore, once mice arrive in a new facility, it is beneficial to allow for a period of acclimation to provide time for their physiological, psychological, and nutritional adjustments to new surroundings. We have routinely allowed 14–21 days for acclimation to 30 °C housing based on empirical observations in our initial studies [54] indicating that this timeframe allows for immunological changes associated with increased anti-tumor immunity to develop. However, there are still sources of variability in environmental stressors associated with particular housing locations within a facility, such as periodic noises from adjacent areas (cage processing, freezer rooms), which can startle the animals and cannot easily be negated. Even if mice are going to be subjected to an imposed stress as part of an experiment, this acclimation period allows for mice in a series of experiments to start the experiments at approximately the same baseline level of stress. Overall, understanding and alleviating these housing stressors as much as possible could lead to both improved animal welfare and improved mouse models of disease. 

## 3. Purposeful Imposition of Experimental Stress for Research

Chronic stress is a pervasive factor in everyday life and is widely associated with negative effects on health, both in the popular press and in scientific/clinical realms. Therefore, in contrast to experiments in which we would like to reduce or eliminate the induction of inadvertent baseline stress, there are a myriad of situations in which stress is imposed on mice to study the effects of chronic stress on both physical and mental disease and to test how stress affects the efficacy of therapies [76]. These include studies in mouse models investigating mechanisms by which stress promotes cancer [77,78,79,80,81,82,83,84,85,86] and therapeutic resistance [45,87,88]. The immunosuppressive effects of stress are also most often studied in mouse models [63,89,90,91,92], including suppression of anti-cancer immune responses [56,61,93,94]. Chronic stress plays a role in aging and immunosenescence, heart disease, obesity, gastrointestinal disease such as ulcers and inflammatory bowel disease [95], and sleep disorders. Additionally, mice are used to study the role of stress in a myriad of neurological conditions such as Alzheimer’s disease [96] and Parkinson’s disease [97]. Mouse models are also used to understand how chronic stress plays into psychological disorders such as anxiety [98], depression [99,100], and Post-traumatic stress disorder (PTSD) [101]. Furthermore, there are ongoing efforts to understand how stress is involved in sociological problems, such as substance abuse [102], the effect of poverty related chronic stress on decision making [103], and the stress induced by noise pollution accompanying urbanization [104]. 

There are several methods for exposing mice to chronic stress. Our lab has studied the effects of stress using the housing temperature model in which mice are continually housed at standard temperatures (stressed) or thermoneutral temperatures to alleviate chronic stress. In contrast, other stress models are achieved by imposing stress on mice housed at standard temperature, and which therefore already have an elevated baseline level of stress [68]. Other stress models that induce continual, chronic stress include manipulation of cage density, for example the social isolation (isolation stress, IS), model where mice, which are social animals, are housed singly [105,106]. In this model, however, singly housed mice cannot huddle for warmth and so may also experience heightened cold stress at the same time. The stress of crowded cages has also been reported to increase tumor growth [107]. There are other approaches in which exposure to stressors is more intermittent. Two models are widely used. The first is restraint stress in which a mouse is placed in a 50 mL conical tube for several hours at a time for several days in a row [80,108]. The second is the chronic unpredictable model of stress (CUMS) in which animals are subjected to several different stressors, including wet bedding, sleep deprivation, foot-shock, cage tilting, water and food deprivation, confinement, continuous illumination, cold stress, and/or forced swim on a random basis [99,109,110]. The CUMS model is thought to be representative of the stressors faced by humans in everyday life and is often used in depression models to study the therapeutic efficacy of candidate anti-depressants; however, a recent study comparing the CUMS to IS found that social isolation is a stronger inducer of depression-like behavior and is more relevant to the role of isolation in stress-induced depression in people [110]. Other models have been developed based on inducing fear, but are not widely used, including exposure of mice to recordings of mice screaming [111], housing adjacent to cats [112], predator odors [113], or chronic social defeat stress (CSDS) in which stranger/aggressor mice are introduced into the cage [101,114]. 

In conjunction with comparing disease progression in control vs. stressed mice, these models are used to test strategies and therapies for reducing or overcoming the effects of stress that can be translated into the clinic. One class of agents which is being very actively investigated is the beta-adrenergic receptor antagonists (β-blockers). The prototypical agent, propranolol, has been widely tested in preclinical studies and shows promising efficacy in overcoming the tumor promoting effects of chronic stress [44,45,56,58,60,87,115,116,117,118,119]. Therefore, there is currently enthusiasm for repurposing β-blockers [120] (currently prescribed for high blood pressure) to overcome therapeutic resistance to chemotherapy, radiation therapy, and immunotherapy. A study providing propranolol off-label to melanoma patients [121] and early randomized clinical trials have reported positive outcomes [120,122,123], validating the rationale provided by the animal studies.

When considering the use of β-blockers in preclinical mouse experiments, we want to point out that these are used as an investigative tool, like β-adrenergic receptor knock-out mice, that have facilitated an understanding of the critical role that adrenergic signaling plays in suppressing the anti-tumor immune response. As such, their use is proving to be an effective pharmacological approach to improving the anti-tumor immune response as an add-on to other therapies in patients. It is important to recognize that other non-pharmacological, bio-behavioral approaches to long-term stress reduction are attractive to patients and are being adopted in survivorship programs to improve quality of life [124,125,126]. 

## 4. How Is Stress Measured, Evaluated, and Compared?

Given the wide range of strategies for inducing stress and the inherent differences in the experimental conditions and parameters used in different labs and experiments, how can we best compare results? Overall, assessments fall into two broad categories, physiological and behavioral. Physiological changes are induced by the sympathetic stress response which prepares animals to react to danger through the well-known “fight or flight” response. Measurable biomarkers include elevated heart rate, increased blood pressure, pupillary dilation, and peripheral vasoconstriction. Stress also induces release of stress hormones such as corticosterone [127,128]. Hickman [129] found that mice exposed to daily intermittent, variable stressors had elevated serum corticosterone (significant in males only) and both male and female mice had elevated neutrophil: lymphocyte ratios; this correlated with results of behavioral tests confirming the stressed status of the mice. Measurement of corticosterone metabolites in fecal material has also been validated as a non-invasive method of monitoring stress over time [128]. Stress also triggers release of neurotransmitters, primarily the catecholamines—adrenalin from the adrenal gland and norepinephrine from sympathetic post-ganglionic neurons. We and others have documented increases in serum catecholamines in chronically stressed mice [44,45,112]. Under chronic stress, animals lose weight and the adrenal glands become enlarged, so reduced body weight and increased adrenal weight are sometimes used to assess stress in experimental groups compared to unstressed/reduced stress controls [43]. Although it was also suggested that overall body temperature, as measured by infrared thermography, could be developed to evaluate stress, Gjendal et al. tested this approach after exposing mice to mild acute stressors (anesthesia, saline injection, scruffing) and did not detect significant effects on body temperature or behavior, but they did not use any chronic stress models [130].

In addition to inducing physiological changes, chronic stress can cause anxiety and depression in mice and is often used to generate models of these diseases. These psychological conditions are reflected by behavioral changes that are monitored by several different tests. Overall, these behavioral changes are “anxiety-like” or “depression-like” in as much as they mirror certain behaviors observed in anxious humans, such as when rodents become hypervigilant, freeze in place and/or exhibit reduced exploratory behaviors, and eat less. The usual experimental design subjects mice to stresses and then evaluates the development of these characteristic behaviors using two or more tests. The elevated plus maze (EPM) test is one of the most widely used. This plus-shaped maze has open and enclosed arms and measures the amount of time a mouse prefers the safety of the closed arms in comparison to being on the open, exposed arms [101]. In the open field test, a mouse is placed in a large enclosure and the amount of time spent against the sides vs. being exposed exploring the open center is measured [101]. The sucrose preference test is often used in depression studies to measure anhedonia and the efficacy of anti-depressant candidates. In this test, mice are given a choice between water and a sucrose solution, which will be preferentially consumed by the control group. As with many of the stress-inducing strategies and assessments, there is variability in reliability and Liu et al. have published a Nature Protocol to improve the reproducibility of results obtained with this test [131]. Other tests which are frequently used are the forced swim test, in which the time it takes for a mouse to “give up” and float is a measure of depression, as well as the tail suspension test which measures immobility (depressive behavior) and activity [100,110]. Overall, the stressors are imposed to generate models of psychiatric conditions and then the efficacy of therapeutic approaches is measured by discerning their ability to counteract these behaviors as evaluated by the various behavioral tests. We recognize thought, that each investigator must decide which stressors and assessments are best suited for their own experiments. One approach to helping with selections is presented in Table 1: some stressors and tests are inherently more stressful than others and selection of combinations that generate the least amount of stress are desirable.

One important caveat in using behavioral tests to evaluate stress is that a mouse is sensitive to the type of handling that is being used [74,75,132,133]. As mentioned above, capturing and picking mice up by the tail method induces anxiety that interferes with interpretation of behavioral tests. For example, in a habituation–dishabituation test, in which mice are presented with urine samples from one mouse in three different exposures and then the 4th time, they are given a novel urine sample from a different mouse, they are expected to show decreasing interest in the first sample over three consecutive exposures and then re-engage with the new sample. However, when the sample is placed in the center of an open field, tail-handled mice are more apt to evidence anxiety by staying in the periphery and not venturing into the center of the space, thus the testing is compromised. In contrast, mice handled by non-aversive techniques will explore the central area (they show less anxious behavior) and are better able to pay attention to the actual test [133] 

## 5. Conclusions

Inherent in our stewardship of animals used for research is the obligation to provide for their welfare and to make every animal count. We should all be concerned about the ability to reproduce results and validate experimental outcomes. Understanding how housing conditions may unintentionally contribute to chronic stress experienced by research mice and thereby compromise interpretations and reproducibility is an important factor in achieving this goal. Furthermore, the use of several different protocols for intentionally imposing chronic stress in mice to study its impact on disease progression is also a potential source of variability. Additionally, we should remember that this additional stress is imposed on whatever baseline stress is already present; this particularly should be considered when interpreting the efficacy of strategies to reduce stress and overcome its effects since these strategies may reduce stress to levels lower than those of the “control” mice which have “baseline stress”. Another question in studying the impact of chronic stress is the choice of the best model. It could be highly beneficial to identify one or two best practice approaches that induce stress without causing unnecessary distress in mice, taking into consideration that some methods of inducing stress may be better for some purposes than others. For example, SI was found to be better than the CUMS for studying depression [111]. Additionally, there are strain differences in baseline levels of anxiety and susceptibility to stressors including chronic unpredictable mild stress (CUMS) as well as in performance in tests measuring behavioral changes associated with level of anxiety or development of depression-like behaviors in mice [101]. Willner [99] states that the differences between C57BL/6 and BALB/c strains, both of which are commonly used in laboratory studies, are the most well studied. Another study of eight different strains, including C57BL/6, showed that BALB/c has a higher sensitivity to CUMS compared to C57BL/6 as judged by a deterioration in the state of the fur in BALB/c used as an indicator of stress [134]. Given the multiple behavioral tests used to assess the effects of stress and pharmacological interventions, it is not surprising that different strains, with different sensitivity to stress, respond differently to both stressors and tests. Jung et al. [135], in a comparison of C57BL/6 and ICR mice, states that following the CUMS, both strains showed increased anxiety in the sucrose and open field tests, but only C57BL/6 showed behavioral changes in the forced swim and novelty-suppressed feeding tests. These examples underscore how important it is for researchers to select the strain(s), protocols for inducing stress, and tests to measure the effects of stress carefully depending on the specific questions they are addressing and to be aware of the strain differences. In fact, differences in stress responses have even been reported between sub-strains of C57BL/6 [136]. 

We do believe, however, that it is important to use a variety of experimental designs and models to increase the robustness and general applicability of the results because whatever strategies show reproducible efficacy in a wide variety of situations and models would seem to have promising clinical potential, although conflicting outcomes can also lead to lack of enthusiasm for a particular approach or agent if the underlying basis for these differences is not appreciated. On the other hand, if an agent is only tested under one set of conditions, its potential efficacy may be missed. Using the example of housing temperature, a chemotherapy may not be effective when the mice are housed at 22 °C but may have significant effects in mice housed at 30 °C [45]. Moving forward, it will be important to provide detailed information about both the housing conditions that contribute to the “undefined vivarium experiment” and the experimental design to maximize the ability to interpret and reproduce results. 

Currently, because there is no one assay for quantifying the degree of stress experienced by a particular mouse, many assessments are used including both measures of physiological biomarkers and subjective behavioral tests. These are adequate for comparisons between groups in the same study, but in the future, it would be helpful to have objective tests to facilitate comparisons between studies. An example would be something that could be measured, such as a blood biomarker, that is not as acutely sensitive to handling stress as stress hormones are. Or perhaps these measures could be stabilized by acclimating mice to the sample procurement procedures. This could also be important since different strains, and even individuals within an inbred strain, have different responses to the same stressor. For instance, an objective measure of stress within a cohort of mice could be critical in understanding the role of stress in the different rates of tumor growth seen within a group of mice. Lastly, an objective, reliable measure of stress may help to compare results of experiments with ones performed under a different set of housing conditions and thus overcome the reluctance to incorporate these basic changes to reduce baseline stress.

## Figures and Tables

**Table 1:** A survey of papers referenced in this review identifies several commonly used procedures for inducing stress (stressors) and commonly used tests for evaluating behaviors induced by stress and/or used to evaluate behaviors associated with psychiatric conditions (behavioral assessments). Here, we provide a subjective comparison of the relative level of stress associated with each procedure alone or in combination with a ehavioral assessment. Investigators should determine which procedures are most appropriate for a particular purpose, but this can provide a guide for selecting procedures minimizing the stress/distress experienced by experimental mice, improving their overall health and experimental outcomes.

Proposed Method for Comparing Degree of Stress Imposed by Experimental Factors			Comparative Stress Levels of Behavioral Assessments	
			1	2
			Sucrose preference, Open field test, Object recognition	Elevated plus maze, Forced swim, Tail suspension
**Stressors, Relative level**	**1**	Social isolation, Restraint (no pressure), Acoustic startle	**2**	**3**
	**2**	Chronic social defeat, Restraint (with pressure) Exposure to predator	**3**	**4**
	**3**	Chronic unpredictable mild stress, Chronic social defeat, Foot shock	**4**	**5**

## Data Availability

Not applicable.
